# Maternal risk factors for abnormal placental growth: The national collaborative perinatal project

**DOI:** 10.1186/1471-2393-8-44

**Published:** 2008-09-23

**Authors:** Kesha Baptiste-Roberts, Carolyn M Salafia, Wanda K Nicholson, Anne Duggan, Nae-Yuh Wang, Frederick L Brancati

**Affiliations:** 1 From the Department of Epidemiology, Johns Hopkins Bloomberg School of Public Health, Baltimore, MD, USA; 2 From Placental Analytics, LLC, Larchmont, NY, USA; 3 From Institute for Basic Research, Staten Island, NY, USA; 4 From the Department of Obstetrics and Gynecology, Johns Hopkins School of Medicine, Baltimore, MD, USA; 5 From the Department of Population and Family Health Science, Johns Hopkins Bloomberg School of Public Health, Baltimore, MD, USA; 6 From the Department of Pediatrics, Johns Hopkins School of Medicine, Baltimore, USA; 7 From the Department of Medicine, Johns Hopkins School of Medicine, Baltimore, MD, USA

## Abstract

**Background:**

Previous studies of maternal risk factors for abnormal placental growth have focused on placental weight and placental ratio as measures of placental growth. We sought to identify maternal risk factors for placental weight and two neglected dimensions of placental growth: placental thickness and chorionic plate area.

**Methods:**

We conducted an analysis of 24,135 mother-placenta pairs enrolled in the National Collaborative Perinatal Project, a prospective cohort study of pregnancy and child health. We defined growth restriction as < 10^th ^percentile and hypertrophy as > 90^th ^percentile for three placental growth dimensions: placental weight, placental thickness and chorionic plate area. We constructed parallel multinomial logistic regression analyses to identify (a) predictors of restricted growth (vs. normal) and (b) predictors of hypertrophic growth (vs. normal).

**Results:**

Black race was associated with an increased likelihood of growth restriction for placental weight, thickness and chorionic plate area, but was associated with a reduced likelihood of hypertrophy for these three placental growth dimensions. We observed an increased likelihood of growth restriction for placental weight and chorionic plate area among mothers with hypertensive disease at 24 weeks or beyond. Anemia was associated with a reduced likelihood of growth restriction for placental weight and chorionic plate area. Pre-pregnancy BMI and pregnancy weight gain were associated with a reduced likelihood of growth restriction and an increased likelihood of hypertrophy for all three dimensions of placental growth.

**Conclusion:**

Maternal risk factors are either associated with placental growth restriction or placental hypertrophy not both. Our findings suggest that the placenta may have compensatory responses to certain maternal risk factors suggesting different underlying biological mechanisms.

## Background

Placental structure and function determine the growth trajectory of the fetus. Several studies show that abnormal placental growth is associated with adverse pregnancy outcomes [1-3]. A disproportionately heavy placenta, suggestive of placental hypertrophy, may indicate an adaptive response to an adverse intrauterine environment. Placental hypertrophy may occur in the presence of conditions such as maternal anemia [4], cigarette smoking [5] and lower socio-economic status [6]. Conversely, a disproportionately small placenta may indicate poor nutrient supply to the placenta, or hypoxia resulting in placental growth restriction and subsequently fetal growth restriction [3].

Effects of individual maternal risk factors on placental weight and its ratio to birth weight have been previously studied [5-12]. Placental weight, however, is a gross summary measure, devoid of biologic and mechanistic detail. Multi-dimensional measures of placental growth may provide further insight into the understanding of underlying mechanisms of fetal adaptation and the gestational intervals in which these processes occur. For example, lateral growth of the chorionic plate plateaus in the middle of the third trimester, while thickness of the placenta increases primarily between 30 and 32 weeks gestation [9].

We are not aware of prior studies identifying maternal risk factors for abnormal placental growth where placental growth is assessed using multidimensional measures of growth. We examined three dimensions of placental growth: 1) placental weight; 2) placental thickness; and 3) chorionic plate area, using data from the National Collaborative Perinatal Project (NCPP). The NCPP is a unique US population-based birth cohort that includes maternal sociodemographic, clinical and obstetric data, gross placental measures and histopathology, infant birth characteristics and childhood growth measures up to age 8. This data provides an excellent opportunity to estimate the influence of maternal characteristics on three dimensions of placental growth in a large population-based sample.

Therefore, our objectives were to 1) determine whether maternal characteristics were associated with multidimensional measures of placental growth and if so, 2) estimate the magnitude of association of maternal factors with placental growth restriction (< 10^th ^percentile in placental weight, thickness or chorionic plate area; and placental hypertrophy (> 90^th ^percentile for placental weight, thickness or chorionic plate area. We hypothesized that lower socioeconomic status (e.g. annual household income, education) would be associated with increased likelihood of placental growth restriction. We also hypothesized that hypertensive disease would be associated with an increased likelihood of placental growth restriction and anemia and gestational diabetes would be associated with increased likelihood of placental hypertrophy.

## Methods

### Study Population

The Collaborative Perinatal Project was a prospective cohort study of pregnancy and child health enrolling participants between 1959–1966. It was specifically designed to identify determinants of cerebral palsy and allied neurological defects [13]. A detailed description of the methods is published elsewhere [13]. Briefly, approximately 42,000 pregnant women were enrolled at 12 hospitals across the United States (Baltimore MD, Boston MA, Buffalo NY, Memphis TN, Minneapolis MN, New Orleans LA, New York NY [2 hospitals], Philadelphia PA, Portland OR, Providence RI and Richmond VA). Pregnant women were usually enrolled at their first prenatal visit. Participants were deemed ineligible if they were incarcerated, were planning to move from the area, planned to give the child up for adoption or gave birth on the day they were recruited into the study. Records were not kept for women who refused participation at baseline.

### Selection Criteria

For the present analysis, eligible mothers met the following criteria: 1) either Black or Caucasian 2) gave birth to live-born singletons, 3) no congenital anomaly, and 4) gestational age ≥ 36 and ≤ 42 weeks.

### Data Collection

Maternal sociodemographic characteristics (age, education, annual household income, smoking status, race) and parity were obtained by self-report during a personal interview. Maternal age was based on age at entry into the study. Education was based on the number of years of education attained and we categorized mothers as having < 12 years vs. 12 or more years of education. Annual household income was collected using categories of $1000 and we dichotomized annual household income using < $5000 vs. $5000 or more (equivalent to $30,000 in 2005). Mothers were classified as non-smokers, light smokers (< 1 pack per day) or heavy smokers (≥ 1 pack per day) at the time of the interview. Obstetrical factors were obtained from clinical examination and laboratory testing. Anemia was defined as having hemoglobin < 10.0 g/dL or hematocrit [Hct] < 30% at any time point during the pregnancy. Mothers were classified as having gestational diabetes if they were diagnosed with diabetes, initiated insulin, received an abnormal glucose tolerance test result, or had a blood glucose ≥ 200 mg/dL during the pregnancy. Hypertensive disease was defined as having a systolic blood pressure ≥ 160 mmHg or a diastolic blood pressure ≥ 90 mmHg and thus includes women with chronic hypertension, pregnancy-induced hypertension and preeclampsia.

Maternal weight and height were measured upon enrollment in the study and mother's weight prior to pregnancy was based on self report. Maternal pre-pregnancy body-mass index (BMI) was calculated as pre-pregnancy weight in kilograms divided by measured height in meters squared. At delivery, just prior to giving birth, mother's weight was measured to determine pregnancy weight gain.

### Placental Measurements

Placentas were collected at delivery and examined by trained pathologists according to a standard protocol [14]. Briefly, the length of the umbilical cord was measured and examined for knots and areas of marked edema. The distance from the base of the cord to the closest placental margin (cord insertion distance) was also measured then the cord was severed at the point of insertion. The largest and smallest diameters were recorded in centimeters and the thickness was measured at the center of the placental tissue by piercing it with a sharp rod calibrated in millimeters. The membranes were then trimmed near the margin of insertion. After removal of the membranes, cord, and any blood clots, the placenta was weighed. We estimated chorionic plate area using the formula for the area [*A*] of an ellipse

$$A=\frac{\pi \cdot d_{L}\cdot d_{S}}{4}$$

where d_L _is the largest diameter and d_S _is the smallest diameter.

### Outcome Measures

Our primary outcomes are the three dimensions of placental growth: placental weight, thickness and chorionic plate area. For each of the three dimensions of placental growth studied–placental weight, placental thickness, and chorionic plate area–the growth dimension was classified as 'restricted' if it was < 10^th ^percentile and 'hypertrophic' if > 90^th ^percentile. Growth dimensions that fell within the 10^th ^to 90^th ^percentile were considered 'normal'. We used the distribution of the placental measures in the NCPP sample to derive the percentiles used in the classification of restriction and hypertrophy.

### Statistical Analysis

Descriptive statistics, frequencies for categorical variables and means and standard deviations for continuous variables were calculated. For each of the three placental growth parameters (weight, thickness, and area), we constructed parallel multinomial logistic regression analyses to identify (a) predictors of restricted growth (vs. normal) and (b) predictors of hypertrophic growth (vs. normal). In multivariate models, we included maternal age, income, education, race, smoking status, pre-pregnancy BMI, pregnancy weight gain, parity, anemia, gestational diabetes and hypertensive disease as covariates. Results were robust before and after adjustment, therefore we present only adjusted odds ratios in the tables. Analyses were conducted using STATA statistical software (version 9.0; Stata Corporation, College Station, Texas). We provided 95% confidence intervals for all estimates. We used a 2-sided Bonferroni-adjusted P value of (0.0045) to test for significance in multivariate models because of the possibility that some associations with the 11 risk factors might have arisen due to chance. The study was approved by the Johns Hopkins Medicine Institutional Review Board.

## Results

Four percent of the subjects enrolled were lost to follow up before delivery. Of 34,345 eligible mothers, 10,921 were excluded from analysis because of missing values for variables of interest. An additional 4 were excluded due to implausible values, leaving an analytic sample of 23,420 mothers. Compared to the participants excluded from the analysis, participants in the analytic sample were slightly older, more likely to be White, and more likely to have annual household income greater than $5000 (equivalent to $30,000 in 2005; all P < 0.05).

Table 1 summarizes characteristics of 23,420 mothers. The mean age of mothers was 24.5 years. Almost half of the mothers were Black and about half did not complete high school. The majority of the mothers had an annual household income less than $5000, equivalent to less than $30,000 in 2005. Almost half of the women were current smokers. By present standards, the women were fairly lean with a mean pre-pregnancy body mass index [BMI] of 22.8 kg/m^2^. The mean weight gain during pregnancy was 9.7 kg, less than the 11–15 kg recommended today for normal weight women. Most of the women (71.5%) had a previous live birth. Anemia was common (21.5%), but gestational diabetes was not (1.6%). About 2% of the women had hypertensive disease.

**Table 1:** Selected Characteristics of 23,420 Mother Placenta Pairs in the Collaborative Perinatal Project.

**Maternal Socio-Demographic Characteristics**	
Age (years)	24.5 ± 5.9
Education (years)	
≥ 12	11,087 (47.4)
< 12	12,333 (52.7)
Race	
White	12,198 (52.1)
Black	11,222 (47.9)
Annual Family Income	
≥ $5,000^†^	6,907 (29.5)
< $5,000^†^	16,513 (70.5)
Smoking Intensity	22.8 ± 4.3
Non-Smoker	12, 350 (52.7)
Light Smoker (< 1 pack/day)	7, 084 (30.3)
Heavy Smoker (≥ 1 pack/day)	3, 986 (17.0)
Pre-pregnancy BMI (kg/m^2^)	22.8 ± 4.3
Pregnancy weight gain (kg)	9.7 ± 5.0
	
**Maternal Obstetric Characteristics**	
Anemia	5,024 (21.5)
Gestational Diabetes	360 (1.6)
Hypertensive disease	543 (2.3)
Parity	
Nulliparous	6,687 (28.6)
Parous	16,733 (71.5)
	
**Placental Characteristics**	
Placental Weight (grams)	438 ± 92
Placental thickness (mm)	21.9 ± 4.8
Chorionic plate area (cm^2^)	247.5 ± 51.3

All results presented as n (%) or mean ± SD

^† ^Equivalent to approximately $30,000 in 2005.

The results of the multinomial logistic regression analysis for placental growth restriction are shown in Table 2. Compared to their counterparts, black women were more likely to have evidence of growth restriction for all three placental growth dimensions examined with relative risk ratios [RRR] ranging from 1.36 to 3.22. Compared to their healthier counterparts, mothers with hypertensive disease were almost twice as likely to produce placentas that were growth-restricted for placental weight and chorionic plate area with RRRs of 1.98; 95% Confidence Interval [CI] (1.54, 2.55) and 1.86; 95% CI (1.46, 2.37) respectively. In contrast, mothers with less than 12 years of education compared to mothers with at least 12 years of education were approximately 20% less likely to have evidence of growth restriction for all three dimensions of growth. Similarly, mothers with an annual household income less than $5000 compared to those with income greater than $5000, were 20% less likely to have evidence of growth restriction for placental thickness. Pre-pregnancy BMI, and pregnancy weight gain both appeared to be protective, showing inverse associations with growth restriction for all three dimensions of placental growth. In addition, mothers with anemia were 33% and 20% less likely to have growth restriction for placental weight and chorionic plate area respectively.

**Table 2:** Multivariate Regression Coefficients for the Association of Maternal Risk Factors with Placental Weight, Thickness and Chorionic Plate Area by Placental Growth

	Placental Growth					
	Placental Restriction (< 10^th ^percentile)			Placental Hypertrophy (> 90^th ^percentile)		
	Weight (g)	Thickness (mm)	Chorionic Plate Area (cm^2^)	Weight (g)	Thickness (mm)	Chorionic Plate Area (cm^2^)

Maternal age (per 5 years)	1.00 (0.99, 1.01)	1.02* (1.01, 1.03)	0.99* (0.98, 0.99)	1.01 (1.00, 1.02)	0.99 (0.98, 1.00)	1.04* (1.03, 1.05)
AnnualFamily Income						
< $5,000 vs ≥ $5,000	0.88 (0.80, 0.98)	0.79* (0.71, 0.89)	0.87 (0.79, 0.96)	1.19* (1.08, 1.32)	0.92 (0.83, 1.02)	1.21* (1.07, 1.34)
Maternal Education						
< 12 yrs vs ≥ 12 yrs	0.83* (0.76, 0.91)	0.78* (0.71, 0.86)	0.87* (0.80, 0.95)	1.09 (0.99, 1.19)	1.15 (1.04, 1.26)	0.94 (0.85, 1.04)
Maternal Race						
Black vs White	1.66* (1.51, 1.82)	3.22* (2.90, 3.58)	1.36* (1.24, 1.49)	0.57* (0.51, 0.63)	0.70* (0.64, 0.77)	0.80* (0.72, 0.89)
Smoking Status						
Light vs Non-smoker	0.92 (0.84, 1.02)	1.03 (0.93, 1.13)	0.94 (0.86, 1.03)	0.95 (0.85, 1.05)	0.89 (0.81, 0.99)	1.09 (0.98, 1.21)
Heavy vs Non-smoker	1.00 (0.89, 1.13)	1.11 (0.98, 1.27)	0.92 (0.82, 1.03)	0.96 (0.85, 1.09)	0.85 (0.75, 0.97)	1.12 (0.98, 1.27)
Parity						
Parous vs Nulliparous	0.89 (0.80, 0.98)	0.98 (0.88, 1.10)	1.06 (0.96, 1.17)	1.25* (1.11, 1.40)	1.32* (1.18, 1.48)	1.09 (0.96, 1.23)
Anemia						
Yes vs No	0.67* (0.60, 0.74)	1.00 (0.91, 1.56)	0.80* (0.72, 0.89)	1.50* (1.34, 1.67)	1.18 (1.05, 1.32)	1.45* (1.30, 1.63)
Gestational Diabetes						
Yes vs No	0.77 (0.51, 1.16)	0.77 (0.51, 1.18)	0.68 (0.45, 1.02)	1.75* (1.33, 2.32)	1.29 (0.94, 1.76)	1.59* (1.18, 2.13)
Hypertensive Disease						
Yes vs No	1.98* (1.54, 2.55)	1.19 (0.91, 1.56)	1.86* (1.46, 2.37)	0.62* (0.46, 0.84)	0.69 (0.50, 0.95)	0.89 (0.66, 1.19)
Pre-pregnancy BMI (per 1 kg/m^2^)	0.91* (0.90, 0.92)	0.98* (0.97, 0.99)	0.93* (0.92, 0.95)	1.10* (1.06, 1.08)	1.05* (1.04, 1.06)	1.04* (1.03, 1.05)
Pregnancy Weight Gain (per 1 kg)	0.93* (0.92, 0.94)	0.99 (0.98, 1.00)	0.96* (0.95, 0.97)	1.07* (1.06, 1.08)	1.03* (1.02, 1.04)	1.05* (1.04, 1.06)

*P < 0.0045 (Bonferroni-adjusted p-value applied for significance testing)

All results presented as Regression co-efficient (95% Confidence Interval)

Predictors of hypertrophy for all three placental growth dimensions included pre-pregnancy BMI, and pregnancy weight gain. For every unit increase in pre-pregnancy BMI there was a 4 to 10% increase in the likelihood of placental hypertrophy. Similarly, for every kg increase in pregnancy weight gain, there was a 3 to 7% increase in the likelihood of placental hypertrophy. Mothers with an annual household income less than $5000 were 19% and 20% more likely to have hypertrophy compared to mothers with incomes $5000 or more for placental weight and chorionic plate area respectively. Mothers with anemia were 50% and 45% more likely to have hypertrophy compared to mothers without anemia for placental weight and chorionic plate area respectively. Compared to nulliparous women, parous women were 25% and 32% more likely to have hypertrophic growth for placental weight and thickness respectively. Mothers with gestational diabetes were 75% and 59% more likely to have hypertrophic growth for placental weight and chorionic plate area respectively. In contrast, after accounting for other sociodemographic factors, black mothers were 20% to 43% less likely than their white counterparts to have hypertrophy of all three dimensions of placental growth.

## Discussion

Most maternal risk factors were associated with either a hypertrophic or restrictive adaptive growth response of the placenta. Black race was associated with an increased likelihood of growth restriction for placental weight, thickness and chorionic plate area, but was associated with a reduced likelihood of hypertrophy for these three placental growth dimensions. We only observed an increased likelihood of growth restriction for weight and chorionic plate area among mothers with hypertensive disease. Conversely, anemia was associated with a reduced likelihood of growth restriction for placental weight and chorionic plate area. Pre-pregnancy BMI and pregnancy weight gain were associated with a reduced likelihood of growth restriction and an increased likelihood of hypertrophy for all three dimensions of placental growth examined.

The relationship between Black race and placental growth is similar to that observed with birth weight. Blacks are more likely to deliver low birth weight infants compared to their White counterparts [15,16] and in our study Blacks have a higher likelihood of restriction for placental weight, thickness and chorionic plate area. Placental growth precedes birth weight, and previous studies show a positive relationship between placental weight and birth weight [17]. Therefore one could propose that the relationship between Black race and birth weight may be mediated by placentation and placental function. Further understanding of the relationship between placental growth and factors influencing placental growth may elucidate understanding of the cause of race disparity with respect to birth weight and other birth outcomes.

In our study, after adjustment for confounding factors, we observed an increased likelihood of placental hypertrophy and a decreased likelihood of placental growth restriction based on placental weight and chorionic plate area in the presence of anemia. However, our findings should be interpreted with caution since we were unable to separate anemia resulting from maternal under-nutrition or chronic disease versus pregnancy-related physiologic anemia. The literature on the relationships between maternal anemia and placental weight, and its ratio to birth weight are inconsistent. On the one hand, placental weight and placental ratio appear to increase with severe maternal anemia and this effect has been attributed to maternal under nutrition [7,12,18-20]. The thought is that fetal hypoxemia develops consequent to anemia and stimulates growth in order to increase surface area of diffusion exchange and thus offset the impaired oxygen transport [5]. However, there is conflicting evidence showing that placental ratio is lower in the presence of severe iron deficiency anemia [20]. Based on our study results, we are unable to make conclusive statements in support either relationship.

In our study we found that gestational diabetes was associated with an increased risk of placental hypertrophy based on placental weight and chorionic plate area. When we examined the distribution of placental growth measures by gestational diabetes status, we observed an upward shift in the distribution for the subpopulation with gestational diabetes. One explanation for this finding is the increase in the proliferation of peripheral villi and an increase in the length of villi and capillaries observed in the presence of gestational diabetes. This may compensate for the reduced perfusion due the increased vascular resistance [21].

Our study has several strengths. First, to our knowledge, this is the first epidemiologic study to examine the association between maternal risk factors and abnormal placental growth defined using chorionic plate area and placental thickness in addition to placental weight. Second, we were able to assess the relationship between maternal characteristics and placental growth in a population-based sample where the placentas were examined using standardized protocol. Finally, the sample size was large providing great statistical precision.

Several limitations of our study deserve comment. The NCPP is a historical dataset from the 1950's and the management of high-risk obstetrical complications has changed substantially over time and cannot be fully adjusted for in multivariate models. However the NCPP represents a large US population-based database that includes maternal variables and detailed placental measurements. Participants excluded from the analysis differed significantly from those included, which introduces some bias. Given the large sample size we suspect this bias is small. Since all women were not routinely screened for GDM during the study time frame, we have likely underestimated the prevalence of GDM in the study sample. We anticipate that an underestimate of GDM prevalence might have limited the power to detect statistically significant associations between GDM and placental growth. Furthermore, the criterion used to define GDM has changed over time which may also have contributed to the low prevalence of GDM observed. Mother's pre-pregnancy weight was obtained by self-report. Although self reported and measured weights tend to be highly correlated [22-25], accuracy may vary significantly according to age and socioeconomic status [23]. In general women tend to underestimate their weight. There was measurement error in the assessment of the placental growth measures. Placental thickness was assessed only at the center of the placenta. There is some variation of placental thickness throughout the placental organ and since thickness represents diffusion conductance, perhaps it would be more representative if thickness was measured at different sites and then a mean thickness calculated. There may be some differential validity in the measurement of largest and smallest diameter with regard to placental shape used in the estimation of chorionic plate area. In addition, our estimate of chorionic plate area assumes that the placenta is shaped as an ellipse; therefore we expect that there may be a slight overestimation.

Finally, we are unable to generalize our results to the general population of today since the sample was enrolled over 40 years ago and was comprised largely of low income African American women. The incidence of maternal risk factors has changed over time. The mothers were leaner and gained less weight during pregnancy than would be expected in a more contemporary cohort.

## Conclusion

Our study results suggest that adverse obstetric conditions are either associated with placental growth restriction or placental hypertrophy, but not both. This implies that the placenta may have specific compensatory responses to adverse maternal obstetric conditions, each with a distinct pathophysiologic mechanism. Further research in a contemporary cohort is warranted to elucidate the biological mechanisms underlying the associations between anemia, gestational diabetes, hypertensive disease, maternal pre-pregnancy BMI, and pregnancy weight gain with abnormal placental growth and to determine whether abnormal placental growth might mediate effect on birth weight and childhood growth.

## Competing interests

The authors declare that they have no competing interests.

## Authors' contributions

KB participated in the conception, design and coordination of the study. KB also performed the statistical analysis, interpreted the results and drafted and revised the manuscript. CMS participated in the conception and design of the study and made substantial contribution to the interpretation of study results and critically reviewed the manuscript. WKN provided extensive contribution to the interpretation of data, drafting the manuscript and revising it critically for important intellectual content. AD made substantial contribution with regard to the acquisition of the data and critically reviewed the manuscript. NYW participated in the design and statistical analysis for the data for the study and was involved in drafting the manuscript. FB participated in the conception and design of the study and provided contribution to the interpretation of data, drafting the manuscript and revising it critically for important intellectual content.

## Pre-publication history

The pre-publication history for this paper can be accessed here:


